# Overlap in drug-disease associations between clinical practice guidelines and drug structured product label indications

**DOI:** 10.1186/s13326-016-0081-1

**Published:** 2016-06-08

**Authors:** Tiffany I. Leung, Michel Dumontier

**Affiliations:** Division of General Medical Disciplines, Stanford University, Stanford, CA USA; Stanford Center for Biomedical Informatics Research, Stanford University, Stanford, CA USA

**Keywords:** Clinical practice guidelines, Drug labeling, Drug therapy, Information storage and retrieval, Chronic disease

## Abstract

**Background:**

Clinical practice guidelines (CPGs) recommend pharmacologic treatments for clinical conditions, and drug structured product labels (SPLs) summarize approved treatment indications. Both resources are intended to promote evidence-based medical practices and guide clinicians’ prescribing decisions. However, it is unclear how well CPG recommendations about pharmacologic therapies match SPL indications for recommended drugs. In this study, we perform text mining of CPG summaries to examine drug-disease associations in CPG recommendations and in SPL treatment indications for 15 common chronic conditions.

**Methods:**

We constructed an initial text corpus of guideline summaries from the National Guideline Clearinghouse (NGC) from a set of manually selected ICD-9 codes for each of the 15 conditions. We obtained 377 relevant guideline summaries and their Major Recommendations section, which excludes guidelines for pediatric patients, pregnant or breastfeeding women, or for medical diagnoses not meeting inclusion criteria. A vocabulary of drug terms was derived from five medical taxonomies. We used named entity recognition, in combination with dictionary-based and ontology-based methods, to identify drug term occurrences in the text corpus and construct drug-disease associations. The ATC (Anatomical Therapeutic Chemical Classification) was utilized to perform drug name and drug class matching to construct the drug-disease associations from CPGs. We then obtained drug-disease associations from SPLs using conditions mentioned in their Indications section in SIDER. The primary outcomes were the frequency of drug-disease associations in CPGs and SPLs, and the frequency of overlap between the two sets of drug-disease associations, with and without using taxonomic information from ATC.

**Results:**

Without taxonomic information, we identified 1444 drug-disease associations across CPGs and SPLs for 15 common chronic conditions. Of these, 195 drug-disease associations overlapped between CPGs and SPLs, 917 associations occurred in CPGs only and 332 associations occurred in SPLs only. With taxonomic information, 859 unique drug-disease associations were identified, of which 152 of these drug-disease associations overlapped between CPGs and SPLs, 541 associations occurred in CPGs only, and 166 associations occurred in SPLs only.

**Conclusions:**

Our results suggest that CPG-recommended pharmacologic therapies and SPL indications do not overlap frequently when identifying drug-disease associations using named entity recognition, although incorporating taxonomic relationships between drug names and drug classes into the approach improves the overlap. This has important implications in practice because conflicting or inconsistent evidence may complicate clinical decision making and implementation or measurement of best practices.

## Background

Clinical practice guidelines provide “recommendations intended to optimize patient care that are informed by a systematic review of evidence and an assessment of the benefits and harms of alternative care options” [1]. Increasingly, guideline developing organizations are expected to produce guidelines based upon a systematic review of evidence relevant to the scope of the guideline; for example, guidelines typically are limited in scope to a single condition, and possibly even to a subdomain of that disease, e.g. screening, prevention, or treatment. High-quality CPGs constitute one of the highest levels of application of evidence-based medicine, based on comprehensive searches and appraisal of the literature, including systematic reviews if available [2]. The U.S. Food and Drug Administration (FDA) provides drug structured product labels (SPLs) for every approved drug. SPLs include structured information such as drug indications, contraindications, and adverse effects. Such labeling is based on data from clinical trials, and evidence about drug effectiveness for specific indications or conditions may be provided. Both CPGs and SPLs are each produced using different and rigorous methodologies, but with common intents of promoting evidence-based medical practices and guiding clinician prescribing decisions. As systematic reviews which form the evidence base for CPG recommendations depend upon well-designed clinical trials and studies of drugs’ clinical effectiveness, and SPLs are produced using clinical trials on drug effectiveness, it follows that the evidence base for CPG recommendations and SPL indications should support similar prescribing practices.

Text mining of biomedical texts is increasingly performed to extract associations from otherwise machine-inaccessible text. Electronic health record documents, such as clinical notes and discharge summaries, published scientific literature, and SPLs are all well-established corpora for text and natural language processing. Dictionary-based named entity recognition (NER) systems and machine learning approaches have been applied to identify entities, including drugs and diseases, in such texts, [3–7] however, to our knowledge, text mining of clinical practice guidelines for these entities had not been done until recently. In a previous study, we applied dictionary-based NER as a text mining method to identify disease co-mentions for common comorbid chronic conditions in chronic disease clinical practice guidelines [8]. We focused on 15 common chronic conditions, including obesity, and 14 of the 15 most prevalent chronic conditions among Medicare beneficiaries: hypertension, diabetes mellitus, hyperlipidemia, stroke, asthma, atrial fibrillation, Alzheimer’s dementia and senile dementias, osteoporosis, chronic obstructive pulmonary disease, depression, chronic kidney disease, heart failure, arthritis, and ischemic heart disease [9]. In that study, ontologies from Stanford University’s National Center for Biomedical Ontologies were compiled into a comprehensive dictionary of disease concepts for the NER task. Initial evaluation yielded reasonable precision and recall with this approach. While annotating biomedical or clinical text is not a novel concept, the current study uniquely examines clinical practice guidelines, a text corpus not previously studied with this approach. Additionally, the current study aims to demonstrate proof-of-concept of evaluating drug-disease associations in clinical practice guidelines.

In previous unpublished work, we constructed a dictionary of drug concepts to use in the NER task of mining pharmacologic treatment recommendations in CPGs. Drug concepts were utilized as a flat list without utilizing available taxonomic information available [10]. We focused on the same 15 chronic conditions to examine how well CPG recommendations about pharmacologic treatment options for the chronic conditions match with SPLs that include one of these conditions as a treatment indication. We found that our recall was low because we did not account for drug classes in the drug-disease associations. We reasoned that differences in the language and structure of CPGs and SPLs may contribute to differences in identified drug-disease associations in CPGs and SPLs. For example, a CPG on heart failure may recommend using an angiotensin-converting enzyme inhibitor, a drug class rather than a specific drug. However, a SPL for a specific drug, such as lisinopril, would specify heart failure as an indication. In this case, *angiotensin*-*converting enzyme inhibitor*-*heart failure* in a CPG drug-disease association should also match a similar drug-disease association in SPLs, such as *lisinopril*-*heart failure*. Simple term recognition methods have an advantage of scaling well to larger datasets with little to no impact on accuracy, compared to advanced natural language processing methods [11]. Given CPG text has not previously been mined before, it was reasonable to apply NER as the initial method for CPG text processing, although the approach has been applied to clinical notes, biomedical literature, and SPLS previously [3–7].

In this work, we use terminologies with structured hierarchies to improve our approach. We utilized the parent-child relationships from the taxonomic structure of a drug classification to find class-based matches for ontologically related terms. To accomplish this, we selected one ontology that had the highest precision when drug names were manually reviewed in a subset of guideline recommendations in the text corpus. We hypothesize that drug-disease associations in CPG recommendations should overlap with drug-disease associations in SPL treatment indications when drug classes and drug names are matched using taxonomic relationships. This would suggest that FDA-approved indications for drugs and guideline-recommended pharmacologic therapies for certain conditions reinforce similar evidence-based drug prescribing.

## Methods

**Fig. 1:**
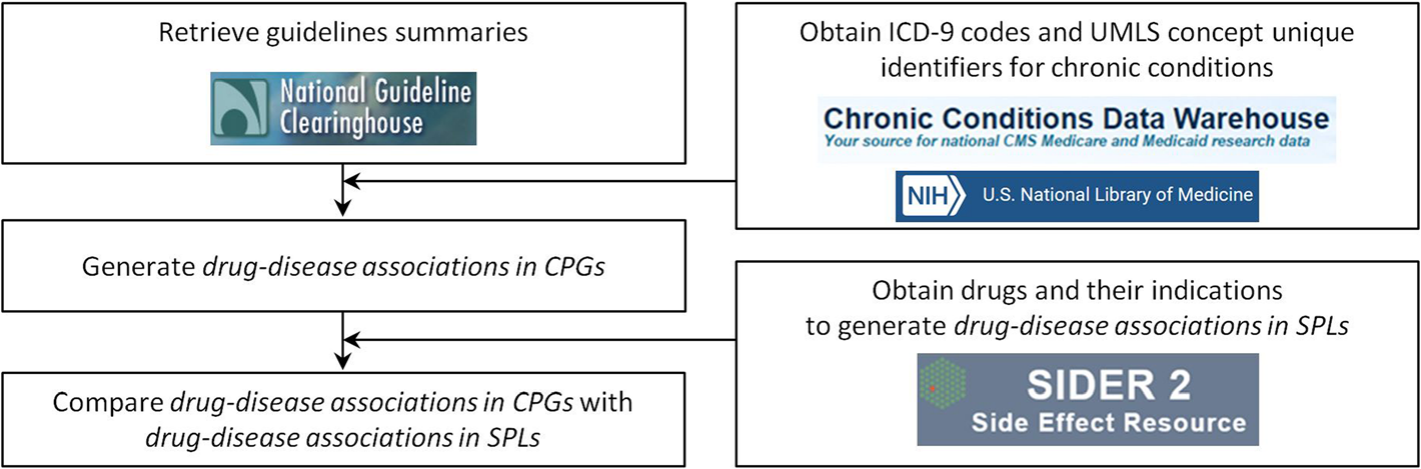
Pipeline for generating and comparing drug-disease associations in clinical practice guidelines and structured product labels

First, we constructed a text corpus containing guideline summaries relevant to the 15 chronic conditions of interest. Then, we created a comprehensive vocabulary of terms for the chronic conditions and performed named entity recognition to identify drug names and drug classes in the text corpus. Next, we performed an evaluation of the method of constructing CPG drug-disease associations. Finally, we compared the overlap between the two sets of drug-disease associations for each chronic condition (Fig. 1). All files used and produced during this study will be available for download at https://github.com/tileung/DrugsInCPGs.

To construct drug-disease associations from the text of CPG recommendations and SPL treatment indications, we apply text mining methods using an expanded set of drug and disease names from multiple terminological resources with taxonomic structure, such as NDF-RT (National Drug Formulary – Reference Terminology) and MESH (Medical Subject Headings), and a data source on drugs, SIDER. A *drug*-*disease association in a CPG* is defined as the occurrence of a drug name mention at least one time in a guideline’s recommendations. A *drug*-*disease association in a SPL* is defined as the occurrence of a chronic condition mention at least one time within the Indications section of a SPL.

### Data sources

We used data and resources from multiple publicly available data sources: (1) guideline summaries from the National Guideline Clearinghouse, (2) drug product label and indication data from SIDER, (3) chronic disease data definitions from the Medicare Chronic Conditions Data Warehouse, and (4) disease and drug ontologies from the National Center for Biomedical Ontology and ABER-Owl Repository [12].

### National guideline clearinghouse

The National Guideline Clearinghouse (NGC), first developed in 1997, identifies published CPGs that meet inclusion criteria and summarizes their highlights across 54 guideline attributes, such as Guideline Title, Major Recommendations, and Target Population [13, 14]. For each guideline, the Major Recommendations section includes summarized key recommendations as indexed by the National Guideline Clearinghouse. Each guideline summary is also tagged with Unified Medical Language System (UMLS) Metathesaurus concepts, identifying “major areas of clinical medicine or health care addressed in the guideline” [15]. The NGC then indexes the guideline summaries on a publicly accessible website for retrieval in multiple formats, including XML and HTML. In June 2014, the NGC implemented a new set of inclusion criteria for guidelines included in the NGC repository [1]. As of September 2015, the NGC featured more than 2400 guideline summaries. NGC guideline summaries, in combination with a comprehensive drug vocabulary constructed in this study, were the source of *drug*-*disease associations in CPGs*.

### SIDER

SIDER is a publicly available resource that interprets and extracts information from text and tables from FDA-approved drugs’ SPLs, identifying side effects and medical conditions in the SPLs using UMLS concepts [15]. Each SPL contains a structured section on Indications, which specifies diseases or clinical conditions for which the drug is FDA-approved for use. SIDER 2 was the source of *drug*-*disease associations in SPLs* in this study.

### Medicare chronic conditions data warehouse

The Centers for Medicare and Medicaid Services provides a research database, the Chronic Conditions Data Warehouse (CCW), of Medicare beneficiaries’ chronic disease care. Chronic conditions are defined by ICD-9 codes in the CCW data dictionary available since 2010 [16].

### BioPortal

The National Center for Biomedical Ontology (NCBO) [17], based at Stanford University, provides online tools for accessing and integrating ontological resources, including BioPortal, a repository of biomedical ontologies. BioPortal contained more than 460 biomedical ontologies as of September 2015. ATC (Anatomical Therapeutic Chemical Classification) was included and obtained from Bioportal because this ontology contains high-level drug classes as well as related drug formulations and ingredients. For similar reasons, NDF-RT was also included, and was obtained directly from the National Library of Medicine. In NDF-RT, certain parent classes and their children were included, specifically, Chemical/Ingredient, External Pharmacologic Class, VA Product, Mechanism of Action, and Therapeutic Categories.

### Aber-OWL repository

Aber-OWL is a framework that consists of an ontology repository, as well as web services that enable ontology-based semantic access to biomedical knowledge [12]. Specifically, additional ontologies and their semantic knowledge were obtained from Aber-OWL, including MESH (Medical Subject Headings), NCIT (National Cancer Institute Thesaurus), and CHEBI (Chemical Entities of Biological Interest Ontology), in order to further expand the drug vocabulary. Only subsets of these ontologies were retrieved. For instance, we restricted the set of MESH terms to subclasses of ‘organic chemicals’, ‘chemical actions and uses’, ‘pharmaceutical preparations’ and ‘polycyclic compounds’. For NCIT, we restricted to ‘drug, food, chemical or biomedical material’. Finally, for CHEBI we restricted the classes to those under ‘role’ and ‘organic molecule’.

### Guideline recommendations text corpus

**Fig. 2:**
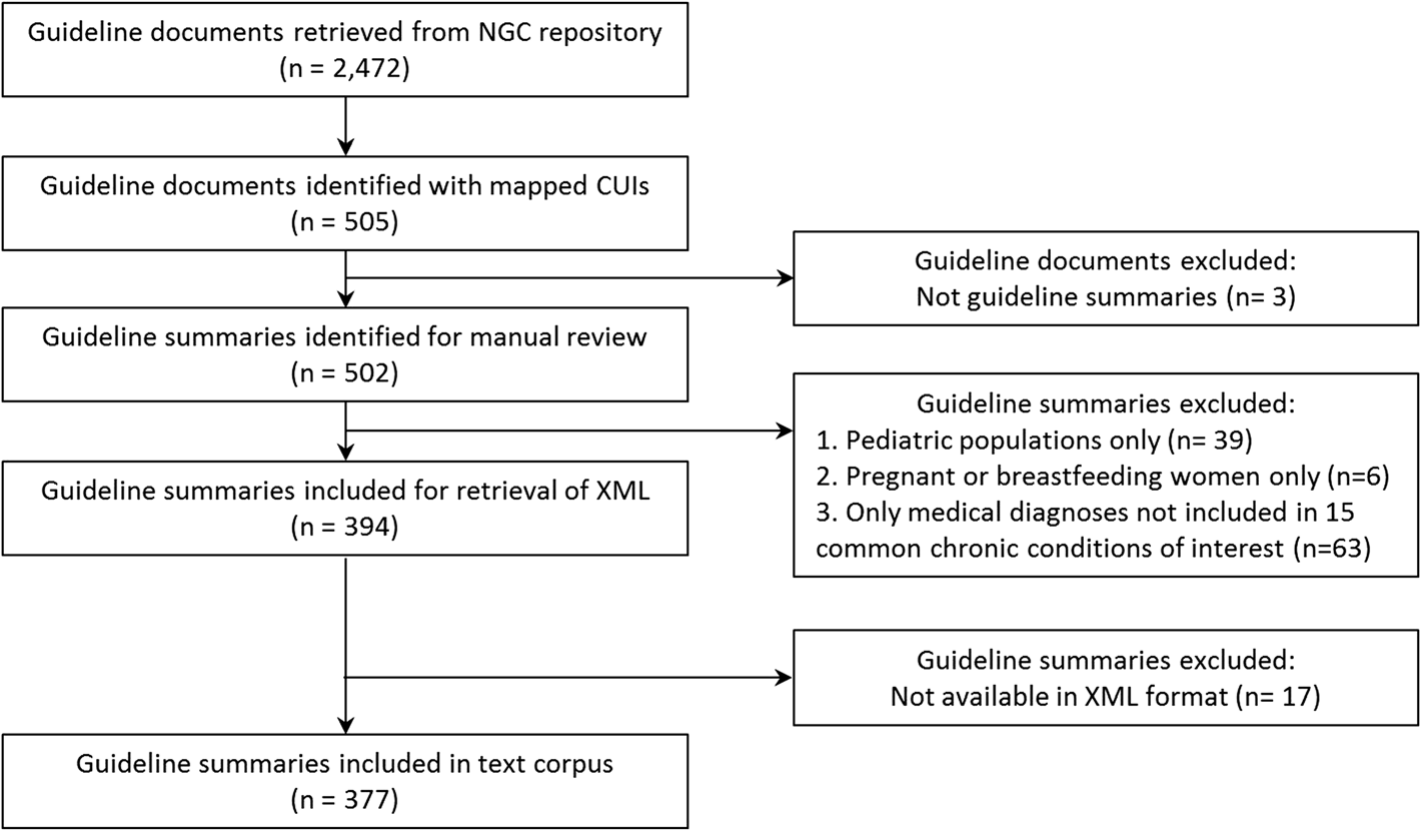
Inclusion diagram for guideline summaries from the National Guideline Clearinghouse

A corpus of guideline summaries was obtained from the website of the National Guideline Clearinghouse. Previously, guideline summaries were obtained from the NGC website in XML format [8], however, an updated text corpus of guideline summaries was constructed in September 2015 because the NGC updated inclusion criteria for guideline summaries. We obtained 445 ICD-9 codes from Medicare CCW data dictionary to identify 14 of the common chronic conditions of interest [9], and added three additional ICD-9 codes for the 15^th^ condition, obesity. The 448 ICD-9 codes representing concepts for the 15 chronic conditions were then mapped to UMLS concept unique identifiers (CUIs). Using the NGC RSS feed, available in XML format, a total of 2472 guideline documents were identified. The mapped CUIs for the 15 chronic conditions were used to identify UMLS concepts tagged to each guideline summary and identified relevant guideline summaries for retrieval and build the text corpus. Initially, 505 relevant guideline documents were identified. Manual review of the retrieved guideline documents revealed that three were expert commentaries, a different type of NGC summary, and these were excluded. Guideline summaries were also excluded from the text corpus if the target patient population for the guideline was *exclusively* pediatric patients, pregnant or breastfeeding women, or for a medical diagnosis that was not among the 15 common chronic conditions. Additionally, 17 guideline summaries were excluded because they were not available in XML format from the NGC website. After exclusion criteria, 377 NGC relevant guideline summaries remained for inclusion (Fig. 2). We extracted the Major Recommendations section from each guideline summary in order to build the text corpus because this section would be the most likely of all sections in the summaries to contain pharmacologic recommendations.

### Text mining for drug names

We constructed a comprehensive drug vocabulary of 97,079 drug names from five ontologies. We performed named entity recognition of these drug names in each of the 377 guideline summaries and identified 1986 unique drug names in the text. We compared the drug-disease associations in CPGs with the drug-disease associations in SPLs. We also examined the overlap between the two sets of drug-disease associations for each chronic condition. To evaluate the approach, a subset of five heart failure guideline summaries were manually annotated with drug names and drug classes to build a reference standard, as there is no existing set of annotated CPGs to perform this evaluation. Additionally, CPGs may have representations of drug names or drug classes that may not appear in alternative clinical or biomedical texts. The approach was evaluated for each of the five ontologies in order to identify one ontology with the highest precision. The selected ontology was then applied to map drug names and drug classes using the existing taxonomic relationships and generate the final set of drug-disease associations in CPGs.

## Results

We identified 1986 unique drug names in the corpus of 377 clinical practice guideline summaries from the National Guideline Clearinghouse. We found 1109 unique *drug*-*disease associations in CPGs*. We identified 533 SPLs with an indication for one of the 15 chronic conditions. We obtained 449 unique *drug*-*disease associations* from the *SPLs*.

### Evaluation

To evaluate the approach of identifying drug names in CPG text, one annotator with medical expertise (TL) manually annotated drug names and drug classes in five guideline summaries for heart failure to construct a reference standard using TextAE, a text annotation client [18]. To guide manual annotation, occurrences of drug names and drug classes that can be prescribed were annotated. For example, in one guideline summary, “For patients with systolic dysfunction (EF <40 %) who have no contraindications: Angiotensin-converting enzyme (ACE) inhibitors for all patients…Digoxin only for patients who remain symptomatic despite diuretics, ACE inhibitors and beta blockers or for those in atrial fibrillation needing rate control,” [19] the concepts *Angiotensin*-*converting enzyme (ACE) inhibitors* and *ACE inhibitors* were annotated drug classes and considered synonymous, *digoxin* was a drug name, and diuretics and beta *blockers* were drug class names. From the guidelines annotated manually, a total of 178 annotations of drug-disease associations were obtained. We compared the manual annotations in the reference standard with the annotations collected from applying the constructed drug vocabulary from each of the five terminologies: ATC, CHEBI, MESH, NCIT, and NDF-RT. Precision, recall, and F-measure were calculated for each terminology in order to identify the most appropriate terminology for the NER task performed on the clinical practice guideline corpus. For this task, a high precision is desirable, where identified drug names and classes using one of the terminologies is more predictive of a true positive identification of a drug name or class in the text. Of the five terminologies, ATC yielded the highest precision of 0.75 and recall of 0.47 (Table 1). For this reason, the taxonomic relationships in ATC were used to perform drug class and drug name matching to produce drug-disease associations in CPGs.

**Table 1:** Evaluation metrics for each drug terminology identifying drug names and drug classes in guideline summaries

	Precision	Recall	F-measure
ATC	0.75	0.47	0.58
MESH	0.5	0.02	0.04
NCIT	0.31	0.32	0.32
NDF-RT	0.25	0.1	0.14
CHEBI	0.25	0.02	0.04

### Drug name and drug class matching

We utilized ATC (Anatomical Therapeutic Chemical Classification), a commonly used drug classification produced by the World Health Organization [20], to identify matches of different drug names to their parent classes. For drugs included in the set of drug-disease associations in SPLs, ATC codes were applied to the drug names if available by using the World Health Organization’s index of ATC codes. The most specific, or descendent, drug name was used to construct drug-disease associations in CPGs*.* For example, *eplerenone (C03DA04) - ischemic heart disease* was a drug-disease association identified in both SPLs and CPGs; however, *aldosterone antagonists (C03DA) - ischemic heart disease* was a drug-disease association in CPGs only and not in SPLs. In this case, these were considered overlapping, or identical, drug-disease associations in CPGs and SPLs because eplerenone is a descendent of the class of aldosterone antagonists. In another example, *pneumococcal vaccines (J07AL) - heart failure* was a drug-disease association in CPGs, and no additional descendants of the class of pneumococcal vaccines were identified. In this case, this was included as a drug-disease association in CPGs only. Additionally, drug names that were descendants of the same parent class were considered similar. For example, *eplerenone (C03DA04) - heart failure* and *spironolactone (C03DA01) - heart failure* are similar because both descendants of the class of *aldosterone antagonists (C03DA)*.

**Fig. 3:**
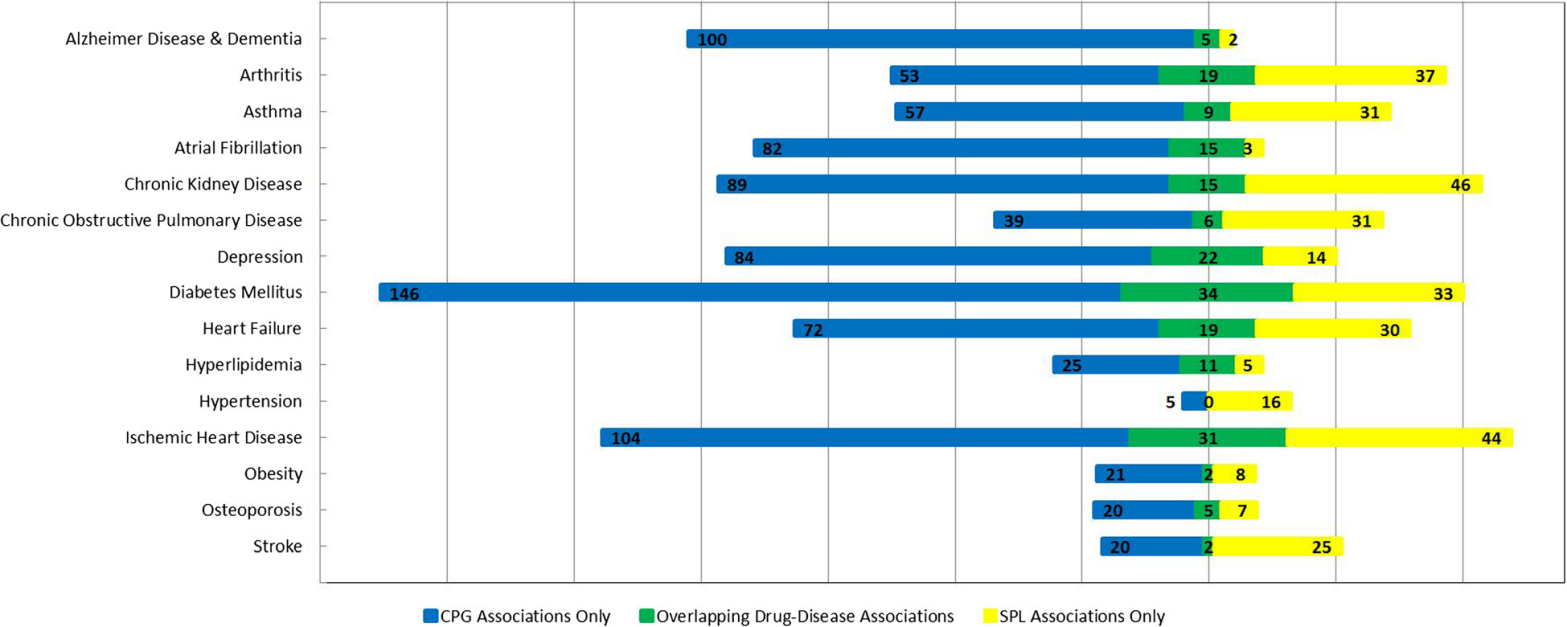
Overlap between drug-disease associations between the two sets of drug-disease associations for each chronic condition, without using taxonomic information on drug names and drug classes

**Fig. 4:**
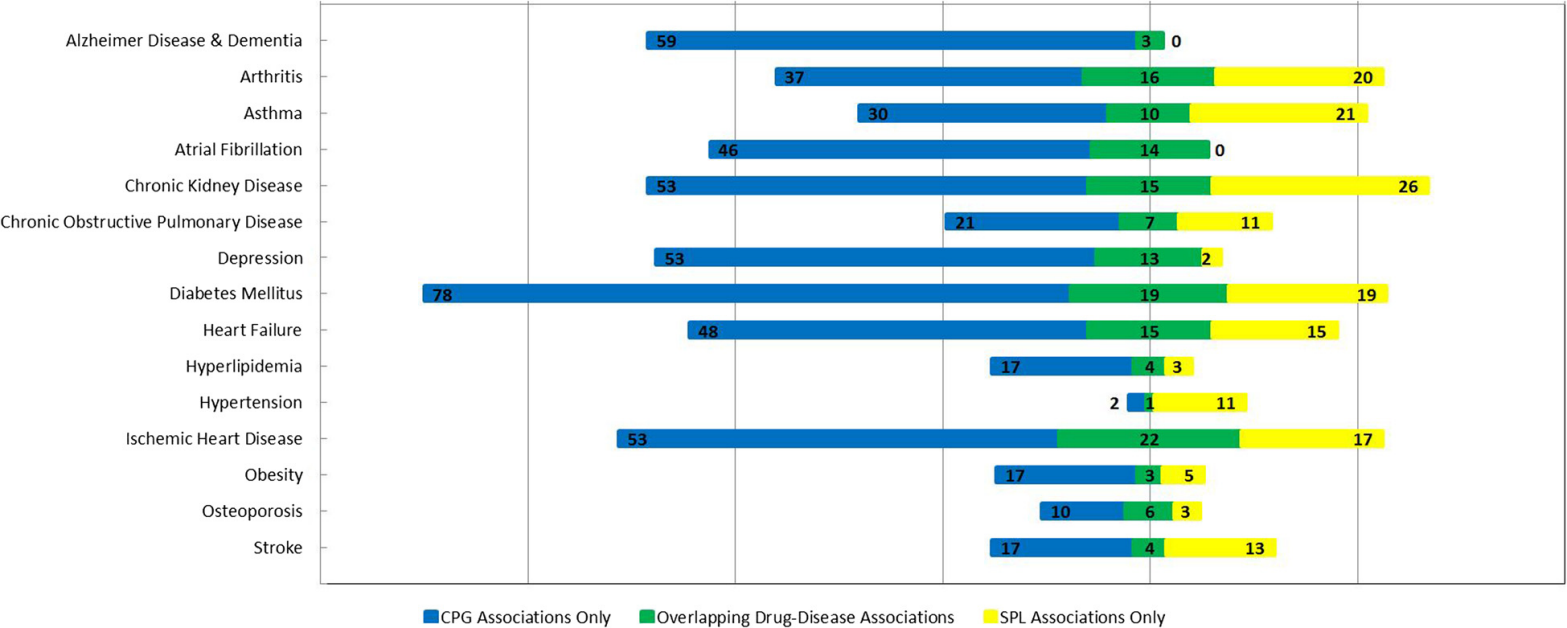
Overlap between drug-disease associations between the two sets of drug-disease associations for each chronic condition, using taxonomic information on drug names and drug classes

In previous work [10], without matching drug names and drug classes, there was minimal overlap between drug-disease associations in CPGs and SPLs for all the chronic conditions (Fig. 3). Without taxonomic information, we identified 1444 drug-disease associations across CPGs and SPLs for 15 common chronic conditions. Of these, 195 drug-disease associations overlapped between CPGs and SPLs, 917 associations occurred in CPGs only and 332 associations occurred in SPLs only. After matching using taxonomic information, 859 unique drug-disease associations were identified across CPGs and SPLs. Of these, 152 of these drug-disease associations overlapped between CPGs and SPLs. This means that CPGs mentioned 541 drug-disease associations that were not also mentioned in SPLs across all conditions; conversely, SPLs mentioned 166 drug-disease associations that were not also mentioned in CPGs across all conditions. The frequency of drug-disease associations in CPGs, SPLs, or both varies depending on which chronic disease guidelines are of interest (Fig. 4).

## Discussion

Our results suggest that guideline-recommended pharmacologic therapies and drug product label indications are reasonably well-matched when taxonomic relationships between drug names and drug classes are incorporated into the text mining approach. Our approach, using both drug names and drug classes, produced superior results over our previous work in which taxonomic relationships were not incorporated into the text mining approach, which resulted in a larger number of mismatches between guideline-recommended pharmacologic therapies and drug product label indications. Overall, the current study demonstrated proof-of-concept that NER, in combination with taxonomic information, can be helpful in identifying drug-disease associations in clinical practice guidelines. It is possible that existing NER and natural language processing systems could be similarly applied out-of-the-box to the text corpus [11]. In clinical practice, knowledge of whether there is consistent and clear medical evidence in both CPGs and SPLs to support certain prescribing practices is informative in the medical-decision making process and personalization of care to the individual patient. Additionally, areas of consistency in the medical evidence in CPGs and SPLs is supportive in the application of such knowledge into best prescribing practices that could be incorporated into clinical information and decision support systems, which is the highest level of application of evidence-based medicine [2].

### Overlapping drug-disease associations

In the approach described here, 541 drug-disease associations were identified in both CPGs and SPLs. An increased overlap between two sources was obtained when information about parent-child relationships between drug classes and drug names was used into the text mining approach. Additionally, the text corpus in this work reflects the most recent NGC guideline summaries as of September 2015, and exclusion criteria were applied to ensure that relevant chronic condition guidelines were included in the study. As of June 2014, the NGC implemented a new set of inclusion criteria to ensure that accepted guidelines provide adequate documentation of their process for systematic review of literature as the basis for the recommendations. This resulted in the retirement of existing guidelines in the NGC repository that no longer met the required criteria. Exclusion criteria appropriately ensured that the included guidelines summaries were applicable to the 15 selected highly prevalent chronic conditions in the Medicare population. As a result of these updates, a corpus of 377 guideline summaries was included.

### CPG drug-disease associations not in SPLs

Our results demonstrate that CPGs contain drug-disease associations that are not also identified in SPLs. One possible explanation is that the natural language of CPGs and SPLs inherently differ, where CPGs may recommend a drug class for a particular condition rather than a specific drug. Additionally, NGC guideline summaries may originate from guidelines produced by professional societies or organizations worldwide, contributing to CPG recommendations for similar drugs of the same drug class. We primarily addressed this limitation by utilizing hierarchical relationships to better match drug-disease associations. However, there remain non-overlapping drug-disease associations. Another possible explanation may be that some CPGs may recommend off-label drug prescribing, which would by definition not be found in SPLs. For example, one CPG recommendation for diabetic neuropathy management states to “offer a trial of duloxetine, gabapentin or pregabalin if a trial of tricyclic drug does not provide effective pain relief” [21]. However, gabapentin does not have a FDA-approved indication for use in diabetic peripheral neuropathy, while duloxetine and pregabalin do have such an indication in their SPLs. Further, it is possible that CPGs may consider utilizing the best evidence available from less robust studies in making recommendations, even though they are intended to be based on systematic reviews of best evidence, which may not be available. In such cases, CPGs typically also convey the strength of evidence for the recommendation. This may mean that CPGs might weakly recommend a certain drug in the treatment of a chronic condition. In contrast, such a process does not exist in the production of SPLs and a SPL for a FDA-approved drug would not suggest prescribing a drug without data on its safety and efficacy from clinical trials. Weak recommendations in CPGs and off-label indications of drugs in CPGs may present opportunities for post-marketing surveillance of the drug for the suggested prescribing practices or may be opportunities for further study towards drug repurposing.

### SPL drug-disease associations not in CPGs

Our results suggest that there are indications from SPLs that are not mentioned in CPGs, even though hierarchical information improved the overlap. One possible explanation is that accurate identification of drug-disease associations in SPLs is necessary. Manual validation of the drug-disease associations in SPLs identified from SIDER would ensure that there is in fact an FDA-approved indication for one of the chronic conditions in each SPL. Another possible explanation is that there may be a delay in integrating the evidence from FDA-approved treatment indications into CPG recommendations. Guideline development can be a prolonged and labor-intensive process, during which new evidence may become available before a guideline’s finalization and approval. This would require further investigation, and may also present important opportunities to streamline the process of implementing medical evidence on the efficacy of newly approved drugs into CPG updates and best practices in clinical medicine.

### Limitations

Our approach is not without limitations. First, a primary objective of this study was to demonstrate proof-of-concept that NER, in combination with taxonomic information, can be helpful in identifying drug-disease associations in clinical practice guidelines. Out-of-the-box NER and natural language processing systems may perform as well as or better than the current approach. Additionally, the evaluation of the CPG text mining method could be more robust – including a larger manually annotated set of guideline summaries, for a variety of chronic conditions and by at least two annotators. Annotating a larger set of guidelines for the evaluation, rather than focusing solely, on heart failure, facilitate a more robust evaluation. Second, utilizing existing annotated clinical or biomedical corpora [4, 22] would allow evaluation of the approach against existing NER tools.

Additional limitations include the guideline repository used to construct the text corpus. Because we utilized the NGC-formulated Major Recommendation section of guideline summaries, these may not contain all the drug mentions found in the full-text documents as published by the original developers. However, if recommendations were made on pharmacologic treatments, then these would likely be identified in the NGC guideline summaries. Accessing and text mining corresponding full text articles will properly assess whether significant differences exist. Additionally, the National Guideline Clearinghouse is the largest available guideline repository that also has a well-indexed, structured, and selective set of guidelines for inclusion. While the current approach was designed to facilitate the process of examining a large corpus of guidelines, after NGC inclusion as well as application of inclusion criteria for this investigation, a relatively small set of guideline summaries remained. Further improvements of the text mining approach may be necessary to ensure accurate information retrieval from the small set of guideline summaries.

Examining drug names and drug classes occurring in text may not be adequate alone. Co-reference resolution, in which a named entity may reference the entity of interest, is a common challenge in text mining and natural language processing tasks and also may impact the current findings. Additionally, we do not extract a precise relationship between a drug mention in a disease CPG. Relation extraction of the context of drug name or class occurrence in the text may better inform the drug-disease association identified [23]. Here, we performed an initial assessment of drug-disease associations in CPGs, and additional methods may better disambiguate the meaning and context of each drug mention in the text. Initial manual examination of a random sample of 20 drug-disease associations yielded seven types of indication relationships (true positives), five other types of relationships (false positives), and two drug-disease association misclassifications. These findings can inform future work improving the text mining approach. Expanding the review to 30 drug-disease associations yielded a higher frequency of all of the indication relationships but did not change the numbers of types of relationships identified on the initial review. Of the 30 drug-disease associations, there were two drug misclassifications where the context of a drug mention in CPG text was not as a prescribable drug (*stroke-oxygen* and *diabetes mellitus-glucose*). Of the remaining 28 drug-disease associations and their source guideline summaries, nine overlapped with drug-disease associations in SPLs. Of the drug-disease associations identified, there were 27 occurrences of seven types of indication relationships, including having an indication: (1) for only the primary disease, (2) for a patient characteristic present with the primary disease, (3) in a specific clinical setting, (4) in combination with another drug for the primary disease, (5) as alternative or non-first-line therapy, (6) for prevention of a comorbid condition, and, most frequently, (7) for the primary disease when another comorbid condition was also present. These relationships represent true positives of drug-disease associations in CPGs. Additional relationships included: drug causes the disease as an adverse effect, drug has a contraindication for the primary disease, drug necessitates additional monitoring requirements in the setting of the disease, and no recommendation can be made about the indication of a drug for a disease. These relationships represent false positives of drug-disease associations. The risk of false positives may be mitigated by additional text processing depending on the relationship extracted, for example, for contraindications, examining the Contraindications section of a SPL may be a useful task. Among the remaining 20 drug-disease associations in CPGs that did not overlap with SPLs, four of the indication relationships were represented (for only the primary disease, in combination with another drug for the primary disease, for prevention of a comorbid condition, and for the primary disease when another comorbid condition was also present). In some cases, the indication was off-label for the primary disease, for example, one guideline on atrial fibrillation states, “Where oral anticoagulants are unavailable, clinicians might offer a combination of aspirin and clopidogrel” [24]. In this case, clopidogrel has FDA-approved indications only for acute coronary syndrome and recent myocardial infarction, stroke or established peripheral arterial disease [25]. Detailed and thorough manual review of a larger set of drug-disease associations would provide additional insight about the types of relationships between drugs and diseases in CPGs, and also would be informative in improving the text mining approach. Although labor-intensive to perform a detailed review manually, this would be an important contribution to this field, as clinical practice guidelines have only recently been used as a corpus for text mining.

Finally, SIDER is a database of curated drug-side effect pairs as the primary database, but may include false positives when examined for drug-disease associations in the indications sections of SPLs. At the time of revised submission of this manuscript for publication, a newer version of SIDER was published that determines its accuracy against other resources and estimates adverse event misclassification [26]. Utilizing the latest dataset of SPLs from SIDER 4 may facilitate more accurate identification of drug-disease associations from SPLs for future work.

## Conclusions

Our work offers a first look at the overlap in CPG and SPL content with respect to drug-disease associations. Our results suggest that CPG-recommended pharmacologic therapies and SPL indications do not overlap frequently when identifying drug-disease associations using named entity recognition, although incorporating taxonomic relationships between drug names and drug classes into the approach improves the overlap. Mismatches between guideline-recommended pharmacologic therapy and FDA-approved drug indications may have a number of implications, including presenting practical challenges in evidence-based clinical practice, such as adding complexity to clinical decision making and implementation or measurement of best practices. Further evaluation and improvement of our methods may be necessary, including examining the relationship between a chronic condition and a drug in a guideline or drug label. Additionally, manual annotation of a larger reference standard or use of existing annotated biomedical or clinical corpora will be relevant to evaluate our approach and how well it performs for each chronic condition. Finally, a detailed manual review of areas where drug-disease associations do not overlap between CPGs and SPLs would be informative, potentially guiding opportunities for further investigation about areas of uncertainty in drug prescribing and their indications. This study is a first step towards further understanding of CPGs and SPLs as congruent resources for evidence-based clinical practice.
